# The nutritional requirements of *Caenorhabditis elegans*

**DOI:** 10.1186/s12263-019-0637-7

**Published:** 2019-05-06

**Authors:** Aleksandra Zečić, Ineke Dhondt, Bart P. Braeckman

**Affiliations:** Department of Biology, Laboratory of Aging Physiology and Molecular Evolution, Ghent University, 9000 Ghent, Belgium

**Keywords:** *Caenorhabditis elegans*, Nutrition, Model organism, Diet

## Abstract

Animals require sufficient intake of a variety of nutrients to support their development, somatic maintenance and reproduction. An adequate diet provides cell building blocks, chemical energy to drive cellular processes and essential nutrients that cannot be synthesised by the animal, or at least not in the required amounts. Dietary requirements of nematodes, including *Caenorhabditis elegans* have been extensively studied with the major aim to develop a chemically defined axenic medium that would support their growth and reproduction. At the same time, these studies helped elucidating important aspects of nutrition-related biochemistry and metabolism as well as the establishment of *C. elegans* as a powerful model in studying evolutionarily conserved pathways, and the influence of the diet on health.

## *Caenorhabditis elegans* ecology and diet in nature

### Habitat

*Caenorhabditis elegans* is a free-living nematode with cosmopolitan distribution [1]. From its first isolation in 1900 by Emile Maupas, this 1-mm-long roundworm was described as a soil nematode. However, the worm is rarely found in pure soil but prefers humid patches that are rich in decaying plant material. It is often found in human-associated habitats, such as botanical gardens, orchards and compost heaps, where it prefers rotting stems, but it can occasionally be found in rotting fruits and flowers [2]. More recently, *C. elegans* has been isolated from forests and scrubland [3–7]. The main characteristic of these semi-natural and natural habitats is that they are rich in microbes and rotting vegetation. The individuals sampled occurred as a specialised larval diapause stage named dauer, which is formed due to absence of food, overcrowding or high environmental temperature, and is a main dispersal stage for colonisation of new food patches, by invertebrate carriers [1, 2]. Interestingly, in certain samples from France [5] and Northern Germany [8], *C. elegans* were found inhabiting the digestive tract of slugs as both dauer and feeding stages, suggesting that these worms can utilise slug intestinal microbes as a food source in the absence of decaying plants.

### Food

In nature, *C. elegans* mainly feeds on different species of bacteria. These include soil bacteria such as *Comomonas* sp., *Pseudomonas medocina* and *Bacillus megaterium* [5, 9, 10]. The most commonly found bacteria in rotting fruits are *Acetobacteriaceae* (*Acetobacter* and *Gluconobacter*) and *Enterobacteriaceae* (*Enterobacter*) and therefore may represent a food source [11]. Moreover, intestinal extracts of freshly isolated *C. elegans* individuals contain also some digested eukaryotes, mostly yeast cells [5]. There is also a possibility that *C. elegans* takes up partially processed plant or animal material, found in decaying vegetation. This may ensure the intake of nutrients that the bacterial food sources cannot provide [1].

### Feeding and food-related behaviour

Food ingestion in *C. elegans* is mediated by the pharynx, a neuromuscular tube-like organ that filters particulate food from a liquid suspension [12], concentrates and grinds it, and transports it further to the intestinal lumen [13], where the nutrients are taken up by intestinal cells. Similar to higher organisms, *C. elegans* is capable of making complex decisions based on the presence and quality of food in their environment [14]; they can learn to seek food which best supports their growth and avoid low-quality food and pathogenic bacteria [14, 15]. Additionally, worms on high-quality food display a behaviour named ‘satiety quiescence’, where they stop feeding and moving and become quiescent [16]. However, males are willing to leave the food in search for a mate [17, 18].

## Dietary choices in the lab

### Commonly used bacterial strains

While information about the *C. elegans* dietary choices in nature is scarce, its diet in the laboratory setting is quite well standardised. In the lab, *C. elegans* is cultured on agar petri plates seeded with bacteria. The most commonly used bacterial food source is the *Escherichia coli* strain OP50, a uracil auxotroph. Its use was advocated by Sydney Brenner as it grows in thin lawns which allow easier visualisation and mating of the worms [19]. Some researchers also use the *E. coli* wild-type K12 strain [20, 21], which forms a thick lawn and can support large worm populations on plate. The K12-derived strain HT115 (D3), which has a disrupted RNAse III gene, is widely used for RNA interference (RNAi) by feeding [22, 23]. HB101, a hybrid between *E. coli* strains K12 and B [24], forms a low-viscosity lawn as the cells do not adhere to each other, facilitating uptake by the worms [9, 25].

### Biomass composition of *E. coli*, *C. elegans* and mammalian cells

Bacterial food provides worms with both macronutrients, which are used as sources of energy and as building blocks, and micronutrients, such as co-factors and vitamins. The average *E. coli* cell is very rich in nitrogen, and its dry weight comprises of approximately 55% protein, 23% nucleic acids (20% RNA and 3% DNA), 7–9% lipids and 6% carbohydrates, while vitamins, co-factors and ions comprise approximately 4% of the dry weight [26, 27]. However, *E. coli* strains used as food source in lab differ in their macronutrient content, especially carbohydrates [28, 29]. For instance, *E. coli* OP50 contains three to five times less carbohydrates compared to HT115 and HB101 [28]. Also, *Comomonas aquatica* DA1877 supports faster growth of *C. elegans* than *E. coli* OP50, due to its higher provision of vitamin B12 [29, 30]. Bacterial strains also differ in the amount of dietary folate or tryptophan they provide to the worms [31–33].

Biomass composition of *C. elegans* has not yet been characterised in detail. Worm dry biomass consists of roughly 60% protein, 20% lipids, 6.5% nucleic acids and 6% carbohydrates [34] (based on data from [35–38]). In mammalian cells, proteins also comprise most of the cell biomass, i.e. approximately 60%. Lipids amount to 13%, nucleic acids to 5% and sugars to roughly 6% of the dry mass [26, 39]. Overall protein and carbohydrate content seem to be similar in *C. elegans* and its *E. coli* food, as well as in mammals. However, relative lipid and nucleic acid content differ considerably between the prokaryotic *E. coli* and the eukaryotic *C. elegans* and mammalian species.

## Nutritional requirements of *C. elegans*

Research into the dietary requirements of free-living nematodes has a long history. In the early twentieth century, Zimmerman reported successful cultivation of *Turbatrix aceti* in an axenic (i.e. bacteria-free) medium for numerous generations. This medium contained glucose, sodium chloride, peptone, lecithin and yeast extract [40].

Pioneering attempts to axenically culture the soil nematode *Caenorhabditis briggsae* highlighted a requirement for a ‘complete’ medium, containing high levels of crude organic components of undefined composition, e.g. chick embryo extract [41], liver [42–44] or plasma [45]. The added organic components contained unidentified, heat-labile protein-like substance(s), commonly named ‘Factor Rb’ [41, 46]. It was initially suggested that this factor was probably a vitamin source [47], but its chemical composition was only determined in subsequent fractionation and supplementation studies [48–52]. ‘Factor Rb’ was also shown to be a required addition to the basic medium [40] for successful growth and reproduction of *T. aceti* [53]. The first completely defined synthetic media for axenic culturing of *C. briggsae* were reported in 1956. The axenic growth medium GM-8 contained D-glucose as an energy source, amino acids, nucleotides, vitamins, salts and growth factors (such as *N*-acetylglucosamine, biotin and ascorbic acid). GM-9 and GM-11 were similar to GM-8 but with omission of certain amino acids and salts [54]. Unsupplemented, these media supported limited growth and reproduction of freshly-hatched larvae, while supplementation with liver medium in traces (0.032% of standard level) resulted in successful growth to adulthood and the production of a limited number of F1 offspring [54]. Subsequent media formulations were based on the composition of basal GM-8 medium (reviewed in detail in [55]).

In 1963, Sayre et al. [56] formulated a defined medium (EM1) based on the amino acid ratio found in *E. coli* instead of casein, as was the case in former media [54]. This medium was further modified by Buecher et al. [57] to a basal medium widely known as the *Caenorhabditis briggsae* Maintenance Medium (CbMM). Discoveries of nematode requirements for sterol source [48, 50], heme [49] and potassium acetate as an energy source [51] revealed the essential components of ‘Factor Rb’. By supplementation of CbMM with these substances, sustained growth of *C. briggsae* in a completely defined medium without added crude extracts was finally accomplished. Slightly modified, CbMM could support continuous growth of other free-living nematode species in axenic culture such as the bacteria-feeding rhabditids *T. aceti* [58–61], *Panagrellus redivivus* [58, 62], *C. elegans* [59, 63] and the fungi-feeding tylenchid *Aphelenchus avenae* [64]. *C. elegans* Maintenance Medium (CeMM) is a modification of the CbMM, which consists of 54 components, with either potassium acetate or glucose (albeit in higher concentration than in CbMM) as energy sources [52], [65–67]. Given that the chemically defined medium preparation is costly and time-consuming, there are additional variants of axenic medium available for cultivating *C. elegans*, one of which contains 3% soy peptone and 3% yeast extract, as sources of amino acids and vitamins, respectively, and 0.5 mg/ml of haemoglobin [68, 69]. Alternatively, *C. elegans* Habituation and Reproduction (CeHR) medium requires addition of milk [70], thus being referred to as semi-defined medium.

## Caloric compounds and building blocks

### Proteins and peptides

Early studies of nutritional requirements of free-living nematodes underlined the absolute requirement for supplementation of axenic media with a heat-labile proteinaceous factor to achieve continuous growth and reproduction [41, 44, 54]. Given that bacteria are food for *C. briggsae* in nature, it was tested whether they could potentially be a source of the proteinaceous factor in a liquid culture medium [47]. For this purpose, *Klebsiella aerogenes* was used: autoclaved and in form of a filter-sterilised supernatant of a cell homogenate. However, both treatments resulted in loss of *K. aerogenes* growth-promoting activity in media consisting of either salts and glucose or ‘Factor Rb’-free autoclaved liver extract [47]. After the discovery that nematodes require an exogenous sterol source, it was reported that growth could be rescued in a medium with autoclaved bacteria and sterol, by adding a liver extract. Fractionation analysis indicated that this heat-inactivated component provided by bacteria and liver is heme [48, 49], which will be discussed in more detail later. With these studies, it became clear that the proteinaceous factor was a source of sterol and heme, which were not provided in any of the chemically defined media [56, 57]. Furthermore, yeast extract also had growth-promoting activity in the form of a pellet of partially denatured ribosomes [71]. This highlighted the importance of particulate matter in a medium and that the growth-promoting activity depends on the uptake, rather than the protein nature of the extract. This was later supported by two studies in which pure precipitated proteins were used [68, 72, 73]. Likewise, *C. elegans* requires particulate matter to successfully take up the nutrients present in axenic medium [74]. By subjecting the medium to 0.22-μm filtration, it retains a very low concentration of small-sized particles that worms cannot ingest but expel with liquid, resulting in impediment of worm growth [74].

It remains unclear from previous studies why tissue extracts still proved to be more efficient in promoting growth in the presence of sterol and heme than any pure protein [72, 73]. A partial answer to this question was provided by a study in which supplementation of medium with glycogen (in the presence of a heme source, but devoid of any protein) had a stimulatory effect on the reproduction of *C. briggsae* [75]. Based on all these findings, Vanfleteren suggested that the growth factor had two roles: to provide the two essential nutrients, sterol and heme and to supply the particulate matter in a medium that would facilitate the uptake of heme [68, 76]. Finally, two studies by Lu et al. revealed that the yet unknown function of the growth factor was to provide an adequate energy source and that it could be fulfilled by adding different lipid components, potassium acetate or glucose [51, 52]. Hence, other than for the heme, *C. elegans* does not require any essential protein or peptide in its diet, unless as a general source of essential amino acids, and these requirements will be discussed in more detail in subsequent sections.

### Amino acids

In 1912, Abderhalden classified amino acids as nutritionally essential and nutritionally non-essential [77]. The former are the amino acids that cannot be synthesised by an animal in normal physiological conditions or not at sufficient rate to support normal growth and thus must be acquired through the diet. On the other hand, non-essential amino acids can be synthesised from precursors in amounts that are sufficient to support the animal’s growth and do not require dietary supplementation. During the evolutionary adaptation to foraging on other organisms, many eukaryotes, including *C. elegans*, lost the ability to synthesise approximately half of the amino acids, in contrast to plants and fungi that are able to synthesise them all [78–81].

Dougherty and Hansen [54] were the first to demonstrate that *C. briggsae* could be successfully cultured in a semi-defined medium that contained arginine, histidine, leucine, isoleucine, lysine, phenylalanine, methionine, tryptophan, threonine and valine as only amino acid supplements. However, added liver extract [54] could have been a source of additional amino acids and/or changed their concentration in the mixture leading to non-reliable conclusions about their essentiality. Later studies on amino acid metabolism based on the use of radioactively (^14^C) labelled precursors showed that *C. briggsae* is capable of synthesising 16 amino acids [82–84]. However, this study could not reveal whether the rate at which these amino acids are synthesised is adequate to sustain growth and reproduction. Finally, Vanfleteren [68, 85] unambiguously showed that the dietary essential amino acids for both *C. briggsae* and *C. elegans* are arginine, histidine, lysine, tryptophan, phenylalanine, methionine, threonine, leucine, isoleucine and valine. The same set of amino acids, except for arginine, represents the dietary essential amino acids for rat and human [78, 86]. Whole-genome sequence analysis by Payne and Loomis [81] indicated that biosynthetic pathways for 10 dietary non-essential amino acids are evolutionarily conserved in rat, human and *C. elegans*. However, the *C. elegans* genome does not encode for orthologues of the enzymes involved in arginine biosynthesis in the human urea cycle (i.e. carbamoylphosphate synthetase 1 (CPS1), ornithine transcarbamoylase (OTC), argininosuccinate synthetase 1 (ASS1) and argininosuccinate lyase (ASL) [81, 87]. In humans, the main source of endogenous arginine is the kidneys. There, citrulline derived from glutamine metabolism in the small intestine is converted into arginine by ASS and ASL and released to blood [87, 88].

### Carbohydrates

An early study of carbohydrate requirements of free-living nematodes reported that fecundity of *C. elegans* cultured in CbMM supplemented with liver growth factor devoid of glucose, inositol and choline was reduced compared to the same medium with added carbohydrates. Addition of 6.5 mg/ml of glucose or trehalose resulted in increased reproduction while supplementation with ribose and sucrose had no such effect [63]. Also, glycogen supplementation to CbMM containing hemin had a positive effect on growth and reproduction of both *C. briggsae* and *C. elegans* [75]*.* In contrast with the previous report [63], addition of trehalose to the hemin-supplemented medium did not support growth and reproduction of nematodes, which was also shown for other carbohydrate supplements such as rice starch, rice flour and corn starch [75]. Overall, these studies did not clearly elucidate the role of carbohydrate supplementation in a defined axenic medium. Lu et al. [51] showed that the addition of potassium acetate to CbMM results in higher population growth of *C. elegans* compared to CbMM supplemented with casamino acids (a casein hydrolysate that contains amino acids and peptides). This was a first study in which a chemically defined compound was characterised as an energy source able to support optimal growth and reproduction in CbMM. Later, it was shown that carbohydrates, in particular glucose and glycogen, and to a lesser extent trehalose (in contrast with [63]), fructose and sucrose, can support *C. elegans* maintenance as a major energy source in axenic medium [52]. Based on this study, it was recommended to increase the glucose concentration of CbMM to 32.5 mg/ml, a level that gives the highest population growth. In a medium without carbohydrate supplementation, worm growth is heavily impaired, which provides definite evidence for the worm’s nutritional requirement for sugar supplementation [52].

In addition to being an important dietary constituent, glucose also has profound effects on *C. elegans* physiology [89–91]. For instance, worms fed a high-glucose diet live shorter and this effect depends on downregulation of the transcription factor DAF-16/FOXO, heat shock factor HSF-1 and the glycerol channel *aqp-1* [91]. This resembles the finding that glycerol channel *Aqp7*-knockout (KO) mice show increased triglyceride levels and develop obesity and insulin resistance when fed a diet rich in fat or sugar [92]. In *C. elegans*, high glucose exerts its detrimental effects through increased formation of reactive oxygen species (ROS), triglyceride accumulation and formation of advanced glycation end products (AGEs) [90, 93, 94]. However, the observed lifespan-shortening effect of a high-glucose diet in *C. elegans* can be alleviated by upregulation of the sterol regulatory element-binding protein (SREBP) homologue *sbp-1* and mediator-15 (*mdt-15*), which induce expression of fatty acid (FA) desaturases, thus decreasing levels of saturated FAs by converting them to unsaturated FAs [95]. Similarly, toxic effects of glucose in mice are reduced by SREBP-induced lipogenesis in hepatocytes, which lowers blood glucose levels [96, 97].

Both in humans and *C. elegans*, excess glucose is stored as glycogen. Primary storage sites of glycogen in humans are the liver and skeletal muscle [98], while in worms, these are the intestine, and to a certain extent the hypodermis and muscle [99–101]. Unlike humans, who possess different isoforms of glycogen synthase in each of the storage tissues [98], *C. elegans* has only one, encoded by *gsy-1* [99, 100]. In non-feeding *C. elegans* dauer larvae increased glycogen levels are important sources of energy for locomotion and nictation behaviour (i.e. dispersal behaviour characterised by upright waving motions) [102]. Moreover, in *C. elegans*, glucose can be stored as the non-reducing disaccharide trehalose via activity of trehalose-6-phosphate synthase, encoded by genes *tps-1* and *tps-2* [103, 104]. Humans, however, do not have this capacity but can break down dietary trehalose [105]. Interestingly, *C. elegans* with reduced *gsy-1* activity live longer and healthier due to elevated storage of glucose in the form of trehalose, in a DAF-16/FOXO-dependent manner [101]. A similar positive effect on lifespan and healthspan is achieved by dietary supplementation of trehalose and inhibition of trehalose breakdown [101].

### Lipids

An isotope labelling study by Perez and Van Gilst [106] revealed that *E. coli* OP50 is the major source of palmitate (C16:0) in lab-cultured *C. elegans.* Additionally, palmitoleate (C16:1ω7), vaccenate (C18:1ω7) and cyclopropane fatty acids (C17 and C19) are also predominantly absorbed from the bacterial food source, although worms can synthesise these fatty acids in very small amounts [106, 107]. The bacterial diet cannot provide stearic (C18:0), oleic (C18:1ω9), linoleic (C18:2ω6) and C20 polyunsaturated fatty acids (PUFAs) [106, 107], but *C. elegans* is enzymatically equipped to synthesise these fatty acids de novo [108, 109]. This ability was first demonstrated for nematodes in the free-living nematode *T. aceti* [110] and later in *C. briggsae* and *P. redivivus* [111] by incorporation of ^14^C-labelled acetate into complete chains of linoleic, linolenic and arachidonic acids in worms grown in axenic culture. Moreover, Lu et al. [51] evaluated the effect of sodium oleate, linoleate and stearate on population growth of *C. briggsae* in CbMM and showed that oleate supported the best growth, potentially due to more efficient utilisation of saturated and monounsaturated FAs than PUFAs by nematodes.

PUFAs are essential constituents of cell membranes conferring their fluidity and semi-permeability. They also have a range of important roles in cell signalling, endocytosis and exocytosis, immune response and pathogen defence [112–114]. The critical step in PUFA synthesis is the production of linoleic acid by desaturases, through introduction of a second double bond into the chain of oleic acid (a monounsaturated FA). This feature has previously been attributed only to plants, fungi and protozoans [115]. However, numerous studies have shown that this step occurs in several insect species [115], certain arachnids [116], pulmonate molluscs [117] and nematodes [108, 110] but not in vertebrates [118]. Mammals lack Δ12 and ω3 desaturases found in plants, hence, plant-derived linoleic and linolenic (C18:3ω3) acids are essential dietary FAs and required precursors for C20 PUFA synthesis [109]. C20 PUFA synthesis in mammals occurs through series of desaturation and elongation steps of essential FAs in the endoplasmic reticulum. These processes are catalysed by Δ6 and Δ5 desaturases and elongases, respectively, to produce arachidonic (20:4ω6) and eicosapentaenoic (20:5ω3) acids [119]. In contrast with plants and humans, *C. elegans* possesses all the enzymes necessary to produce arachidonic and eicosapentaenoic acids. The Δ12 and ω3 desaturases found in plants are encoded by *fat-2* and *fat-1*, respectively, with *fat-1* having both desaturase functions [120–122]. The *C. elegans* homologues of human Δ6 and Δ5 desaturases and ω3 and ω6 elongases are *fat-3, fat-4*, *elo-1* and *elo-2*, respectively [108, 109, 123].

Monomethyl branched-chain fatty acids (mmBCFA) C15ISO and C17ISO represent another group of FAs that are essential for *C. elegans* growth and development and are synthesised de novo [124, 125]. Unlike the biosynthetic pathway of straight-chain FAs, where the primer is acetyl-CoA [126], biosynthesis of mmBCFAs utilises primers derived from branched-chain amino acids (BCAA; leucine, isoleucine and valine), while the chain extender for both pathways is malonyl-CoA [127]. Primer synthesis is catalysed by the branched-chain α-ketoacid dehydrogenase (BCKDH) multi-subunit complex and elongation of the mmBCFA backbone is performed by elongases ELO-5 and ELO-6 [124]. Disruption of BCKDH in humans causes a metabolic disorder named maple syrup urine disease (MSUD), which symptoms are mainly attributed to accumulation of BCAA [128, 129]. Loss of function of *dbt-1* (encoding for the *C. elegans* homologue of the human BCKDH E2 subunit) results in embryonic lethality and arrested larval development, predominantly due to lack of mmBCFAs rather than the accumulation of BCAAs [130]. Hence, this may give new insights into the importance of mmBCFAs for human health.

Fats are sequestered in lipid droplets, organelles that are evolutionarily conserved in *C. elegans* [131], *D. melanogaster* [132], *S. cerevisiae* [133] and mammals [134]. Unlike humans and other vertebrates, *C. elegans* does not possess specialised cells called adipocytes [135], which are the main constituents of the adipose tissue, an organ with endocrine and immune functions [136, 137]. Instead, *C. elegans* stores fats in the intestine, which is a major energy storage organ, similar in function to the liver and adipose tissue. To some extent, fat also accumulates in the hypodermis [131, 135] and muscles [138]. Like glycogen, triglycerides are accumulated in large amounts in *C. elegans* dauer larvae, where they serve as a primary energy source for starvation survival [139, 140].

### Small organic compounds

The ability of free-living nematodes to utilise two-carbon compounds (e.g. acetic acid and ethanol) as an energy source in axenic medium was first demonstrated in three rhabditid species: *C. briggsae*, *T. aceti* and *P. redivivus* [141–143]. Supplementation of ethanol, n-propanol and potassium acetate has stimulatory effects on population growth of *C. briggsae* in CbMM [51]. These short carbon-chain compounds can be readily utilised by free-living nematodes, including *C. elegans*, due to the presence of a set of enzymes for conversion of alcohols into acetyl-CoA, i.e. alcohol dehydrogenase, aldehyde dehydrogenase and acetyl-CoA synthetase [144]. Starved L1 larvae of *C. elegans* live twice as long when treated with 1 mM ethanol. The underlying mechanism is not clear [145] but likely involves conversion of ethanol into acetyl-CoA. This could be an important strategy for starvation survival in harsh environmental conditions.

The fully functional glyoxylate shunt allows *C. elegans* to convert acetyl-coA into succinate, which can fuel the citric acid cycle and the resulting excess of oxaloacetate may be utilised for gluconeogenesis [146]. In addition to nematodes, an operational glyoxylate cycle is present in bacteria, fungi, plants and protozoans, but not in vertebrates. However, some studies reported the activity of one or both glyoxylate cycle enzymes, isocitrate lyase (ICL) and malate synthase (MS), in different tissues of amphibians [147], birds [148] and mammals [149]. Moreover, a comparative genomic study identified a tandem of ICL and MS genes in a cnidarian genome [150]. No study so far managed to find a fully functional glyoxylate cycle in any metazoan besides nematodes.

## Vitamins

Vitamins are defined as a group of organic compounds essential in small amounts for organismal function that cannot be synthesised by the body and thus must be provided through the diet (or via the biosynthetic activity of intestinal bacteria). Unlike macromolecules that are classified based on similarities in their chemical properties, vitamins are grouped based on the function they serve. Different organisms vary in their capacity to synthesise these organic compounds. Hence, what is considered a vitamin for one organism might not serve that function for the other.

### Sterols

Early studies into the dietary requirements of free-living nematodes and attempts to formulate a chemically defined medium that would support growth and reproduction gradually resulted in a defined medium (CbMM) that required supplementation with crude substances for sustainable cultures [54, 56, 57]. One of the essential growth factors that CbMM lacked and the crude extracts could potentially provide were sterols [57]. A study on *Steinernema feltiae* was the first one to report the requirement of sterols for growth and reproduction of nematodes in axenic medium [151]. The same requirement was later demonstrated for *C. briggsae*, *T. aceti* and *P. redivivus* [48, 152]. Parallel biochemical studies revealed that these species are unable to synthesise sterols de novo from ^14^C-labelled acetate and mevalonate [153, 154].

In order to find the missing step(s) in nematode sterol biosynthesis, Lu et al. added five sterol precursors (acetic acid, DL-mevalonic acid lactone, farnesol, squalene and lanosterol) and cholesterol to *T. aceti*, *C. briggsae* and *C. elegans* populations cultured in CbMM [50]. Supplementation with acetic acid, DL-mevalonic acid lactone and farnesol did not have any significant effect on population growth while squalene, lanosterol and cholesterol showed dose-dependent positive effects. Hence, it was proposed that the metabolic block in de novo sterol biosynthesis in nematodes likely occurs between farnesol and squalene and at any of the steps prior to farnesol synthesis [50]. However, these conclusions were in contrast with a previous study reporting that squalene had no effect on growth and reproduction of *E. coli*-fed *C. elegans*. Also, another study indicated no effect of lanosterol on *C. elegans* development in a chemically defined medium [48, 155]. On the contrary, *C. elegans* growth is fully supported only by exogenous provision of ergosterol, β-sitosterol, stigmasterol or cholesterol, which are the final products of sterol biosynthesis in plants and mammals [48, 66]. Indeed, the *C. elegans* genome encodes for homologues of mammalian enzymes involved in the initial steps of sterol synthesis (up to farnesyl pyrophosphate), but no downstream enzymes, including squalene synthase and squalene cyclase [156, 157]. Unlike nematodes, mammals possess a complete set of enzymes required for de novo synthesis of sterols from acetyl-CoA, under the tight regulation of SREBP and thus do not require exogenous sterol supplementation [158].

Under standard laboratory conditions, *C. elegans* sterol requirement is fulfilled by addition of cholesterol to the culture, even though it was shown not to be the essential dietary sterol for *C. elegans* [159, 160]. Similarly, in nature, bacterial food cannot provide dietary sterols; thus, this requirement is likely met by feeding on decaying plant or fungal material or on animal faeces [1, 156].

In mammals, cholesterol is an important component of cellular membranes required for their fluidity and semi-permeability and a precursor for the synthesis of bile acids and steroid hormones. In *C. elegans*, its role is not entirely clear. Given that *C. elegans* membranes contain almost no cholesterol, its structural role was proposed to be less likely. Instead, it was suggested that it could have roles in cellular signalling, related to moulting and dauer formation [156, 160, 161]. Indeed, *C. elegans* uses cholesterol as a precursor for the synthesis of dafachronic acids (DAs), bile-like steroids that bind to the nuclear receptor DAF-12, a homologue of pregnane-X and vitamin D receptors in vertebrates that controls dauer entry and modulates lifespan [162–165]. The key role in DA synthesis belongs to *daf-9*-encoded cytochrome P450, of which the human homologue is sterol 27-hydroxylase (CYP27A1) known to be involved in bile acid synthesis in the liver [163, 166, 167].

### Heme

Supplementation of chemically defined axenic media with organic substances could possibly provide nematodes with yet another essential growth factor which these media lacked: heme [54, 56, 57]. *C. briggsae* can be indefinitely cultured in a salt-buffered medium containing only live *E. coli* supplemented with sterols [48]. When bacteria are heat-killed in such medium, *C. briggsae* cannot grow, suggesting that a certain essential component had been destroyed by autoclaving bacteria. However, *C. briggsae* can be successfully cultured in a medium with autoclaved *E. coli* and sterols supplemented with a heated liver extract that contains heme. The same effect was achieved by supplementation of cytochrome c, haemoglobin, myoglobin or hemin chloride [49]. Addition of hemin chloride to CbMM and EM1 allowed for repeated subculturing of four free-living nematode species—*T. aceti*, *P. redivivus*, *C. briggsae* and *C. elegans*—but the final population size was small, unless the concentration of hemin chloride was high (250 μg/ml) or it was provided in an adequate precipitated form at lower concentration (50 μg/ml) [76, 168]. Under standard axenic culture conditions, the dietary requirement for heme is met by supplementation of the medium with haemoglobin [68, 69] or cytochrome c [66]. Standard culturing on agar plates seeded with *E. coli* does not require addition of heme to the medium, since it is provided by the live bacteria. *C. elegans* utilises dietary heme to provide this prosthetic group to endogenous heme proteins and potentially as an iron source in conditions of environmental iron deficiency [169].

Genome sequence analysis indicated that *C. elegans* lacks orthologues of all mammalian genes involved in synthesis of heme from δ-aminolevulinic acid. These enzymes are cytosolic δ-aminolevulinic acid dehydratase, porphobilinogen deaminase, uroporphyrinogen III synthase, uroporphyrinogen decarboxylase, mitochondrial coproporphyrinogen oxidase, protoporphyrinogen oxidase and ferrochetalase [169, 170].

Being a heme auxotroph, *C. elegans* has been a powerful model to study mechanisms of heme uptake, transport and homeostasis that are evolutionary conserved between worms and mammals [171–176]. Uptake of dietary heme in *C. elegans* is mediated by the activity of the two heme transporters, HRG-1 (heme-responsive gene 1) and HRG-4, which import heme into the intestine [171, 174]. Subsequently, heme is delivered to other tissues and the embryos by the secreted transporter HRG-3 and to the hypodermis by the transmembrane transporter HRG-2 [173, 175]. Finally, the multidrug resistance protein MRP-5 is the key player in regulating heme homeostasis in *C. elegans* by acting as a heme exporter, which is genetically conserved between worms, yeast, zebrafish and mammals [176].

### Other vitamins

First insights into the vitamin requirements of nematodes emerged in the 1950s in a study in which was shown that continuous growth of *C. briggsae* in axenic medium containing autoclaved liver extract could be accomplished by the addition of folic acid [177]. This finding was later confirmed in experiments with aminopterin, a folic acid antagonist, which had severe effects on *C. briggsae* growth and development due to thymine deficiency [178]. In addition to being important for thymine biosynthesis, folic acid is also required by *C. briggsae* for histidine catabolism [178, 179]. These findings were not surprising, given that a biochemical pathway for de novo folate synthesis is present only in plants and microorganisms, while animals require dietary folate to maintain physiological functions. In humans, folate deficiency leads to neural tube defects during embryogenesis and dietary supplementation of folic acid has proven successful in decreasing the occurrence of such birth defects [180, 181]. *C. elegans* feeding on *E. coli* that are mutant in folate biosynthesis show a lifespan extension of 30–50%. However, this is not a consequence of changes in folate uptake in *C. elegans* but probably occurs due to reduction in toxin-based virulence of *E. coli*, related to excess folate that these bacteria produce [31, 33]. In humans and other mammals, folate is taken up by the reduced folate carrier (RFC), which is similar to the folate uptake in *C. elegans* via the carrier encoded by *folt-1* [182, 183].

In addition to folate, all the other vitamins of the B complex were also reported to be essential for normal growth and development of *C. briggsae* in axenic medium: riboflavin, thiamine, pyridoxine, niacinamide, pantothenic acid, biotin and cobalamin [55, 184–186]. Among these, cobalamin (vitamin B12) was most extensively studied in the *C. elegans* model system [30, 187–189]. This vitamin has the unique property of being synthesised solely by archaea and bacteria [190]. However, the common food source for *C. elegans* in the lab, *E. coli*, lacks the vitamin B12 biosynthetic pathway. Hence, worms take it up by ingesting bacteria that absorb the vitamin from the culturing medium [191]. Vitamin B12 deficiency was shown to severely impair *C. elegans* biology, leading to growth retardation, lifespan shortening and reduced egg-laying capacity, which is consistent with results obtained in mice and humans [187, 192, 193]. Also, memory retention in *C. elegans* is impaired, partially due to severe oxidative stress [188]. MRP-5, previously shown to be the heme exporter through the intestinal membrane to other tissues, was also identified as the exporter of vitamin B12 from the hermaphrodite intestine to the embryos [176, 189]. This finding can be explained by the structural similarity of vitamin B12 and heme; both contain a protoporphyrin ring with a cobalt or iron ion in its centre, respectively. Additionally, based on sequence homology, MRP-5 is likely a functional ortholog of the human MRP1 [189]. Apart from vitamins of the B complex, no information is available on *C. elegans* nutritional requirements for other vitamins.

## Minerals

Dietary minerals represent a class of inorganic nutrients that are essential for many metabolic and physiological processes in the body, usually required in small amounts naturally found in different types of food. Minerals are required for signal transduction, maintenance of osmotic balance and acid-base equilibrium, energy metabolism, enzyme functions and, in vertebrates, for formation and maintenance of bones [194]. Importantly, certain minerals are required by all animals, but the amounts in which they must be provided can vary greatly depending on the species and the function of the mineral.

In humans, dietary minerals are classified into principal or macroelements and trace or microelements. The former are required in amounts greater than 200 mg per day and comprise of calcium, phosphorus, sulphur, potassium, chlorine, sodium and magnesium. The latter are required in minute amounts; represent only 0.02% of the total body weight; and include zinc, iron, chromium, copper, cobalt, manganese, molybdenum, selenium, iodine and fluorine [195]. Importantly, both mineral deficiency as well as excess mineral intake can have detrimental effects on health. For instance, iron deficiency can result in anaemia and problems with the immune system, while excess intake causes liver damage [195].

Mineral deficiency studies in nematodes were difficult to perform until a completely chemically defined medium was developed [51]. Deficiency of individual minerals was achieved by depleting them from the basal medium. After depletion, different concentrations of each mineral were added to the medium to determine the quantitative requirements of *C. elegans* [196]. Potassium and magnesium were shown to be absolutely essential minerals for *C. elegans* since no growth was observed when these minerals were removed from CbMM. *C. elegans* could survive copper, calcium and manganese depletion. However, complete deficiency of these minerals was difficult to induce. Similarly, sodium was present in different components of the medium and effects of its absolute deficiency could not be tested. Hence, the authors suggested optimal concentrations of these minerals in CbMM to achieve maximal population growth but gave no conclusion on their essentiality [196]. Moreover, this study could not provide any evidence for the requirement of zinc in *C. elegans*, nor could it create iron deficiency, given that heme was an essential part of the CbMM. A recent study on zinc deficiency in *C. elegans* revealed that lack of zinc reduces worm fertility by causing aberrations in oocyte development and meiotic division [197]. These results are consistent with the role of zinc in mammalian gametogenesis where it is involved in the production of sperm and maturation of oocytes. This establishes *C. elegans* as a suitable model for the study of zinc as a factor in animal fertility [198, 199]. Furthermore, reducing zinc levels in vivo has been shown to extend *C. elegans* lifespan and reduce age-related protein aggregation, partially by inducing DAF-16/FOXO nuclear localization [200].

## Concluding remarks

**Fig. 1:**
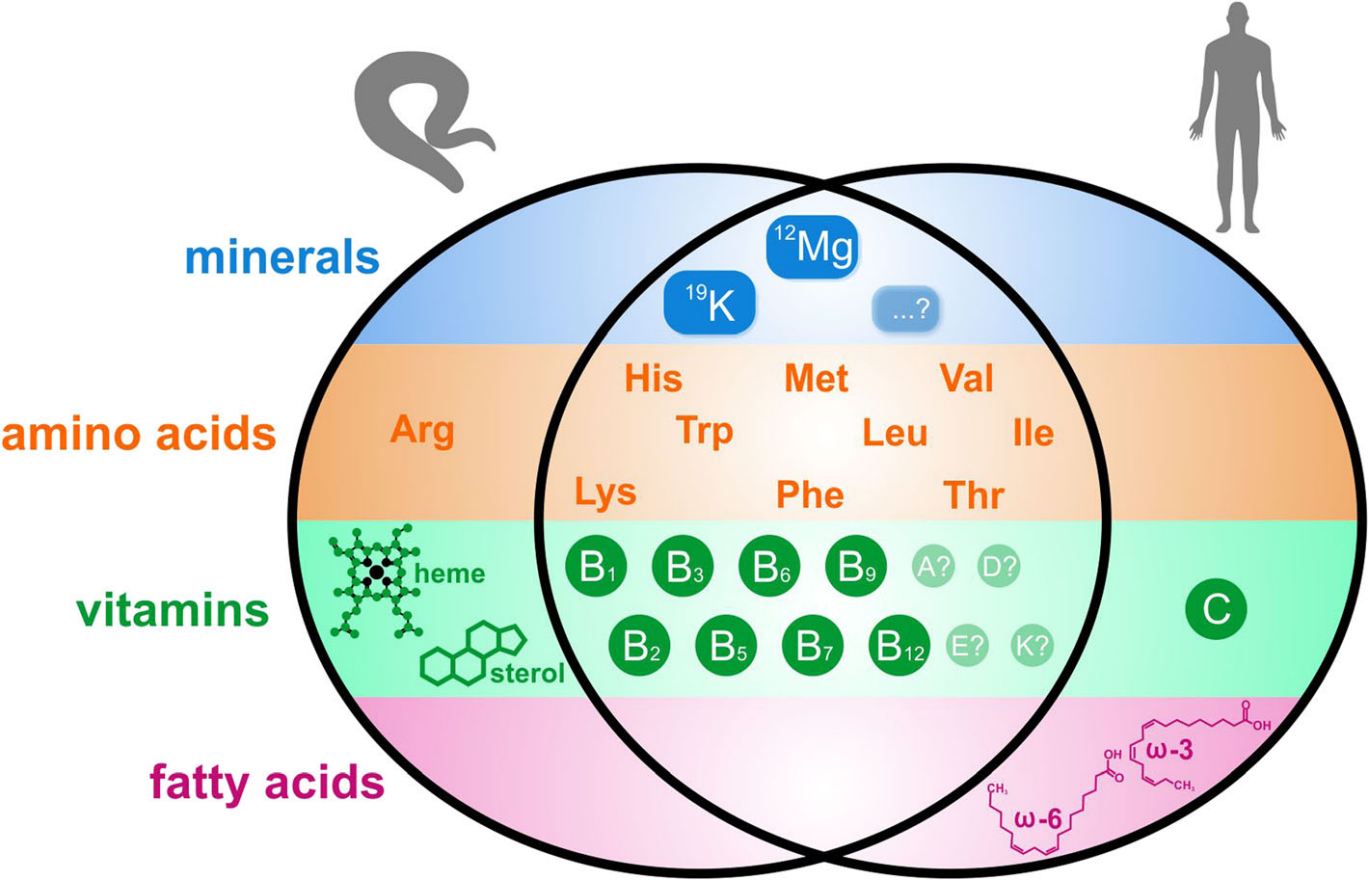
Known common and specific essential nutrients of *C. elegans* and humans

For decades, *C. elegans* has been a preferred model organism to study fundamental metazoan biology, due it its genetic amenability, inexpensive maintenance, easy experimentation and fully annotated genome encoding for homologues of many human disease-associated genes. Therefore, it can also be considered a suitable model for the genetics of animal nutrition and metabolism. Indeed, many dietary requirements and metabolic responses are evolutionarily conserved, such as the fat accumulation as a result of decreased Ins/IGF-like signalling. Yet, one should be well aware that nematodes and vertebrates do not share all enzymatic pathways and thus show some important differences in their dietary requirements. The most profound differences are sterol and heme auxotrophy of nematodes and the types of essential amino acids and fatty acids required by worm and human (outlined in Fig. 1).
